# *In Vivo* magnetic resonance imaging of xenografted tumors using FTH1 reporter gene expression controlled by a tet-on switch

**DOI:** 10.18632/oncotarget.12519

**Published:** 2016-10-08

**Authors:** Xiaoya He, Jinhua Cai, Hao Li, Bo Liu, Yong Qin, Yi Zhong, Longlun Wang, Yifan Liao

**Affiliations:** ^1^ Department of Radiology, Children's Hospital of Chongqing Medical University, Chongqing, China; ^2^ Ministry of Education Key Laboratory of Child Development and Disorders, Chongqing, China; ^3^ Key Laboratory of Pediatrics in Chongqing, Chongqing, China; ^4^ Chongqing International Science and Technology Cooperation Center for Child Development and Disorders, Chongqing, China

**Keywords:** magnetic resonance imaging, ferritin heavy chain, tetracycline-inducible expression system, cell tracking, tumor xenograft

## Abstract

As a promising magnetic resonance imaging (MRI) reporter, ferritin has been used to track cells *in vivo*; however, its continuous overexpression can be cytotoxic, which restricts its application. In this study, we aimed to develop a switch to turn this genetic reporter “on” or “off” while monitoring cell grafts via MRI. To accomplish this, we genetically modified the ferritin heavy chain (FTH1) with a Tet-On switch and assessed the expression of FTH1 in transduced neuroblastoma cells (SK-N-SH) *in vitro* and in xenografted tumors *in vivo*. We found that FTH1 expression induced by doxycycline (Dox) in SK-N-SH-FTH1 cells depended on treatment dose and duration. We successfully detected T_2_-weighted MRI contrast in cell grafts after switching “on” the reporter gene using Dox, and this contrast disappeared when we switched it “off”. The genetic reporter FTH1 can thus be switched “on” or “off” throughout longitudinal monitoring of cell grafts, limiting expression to when MRI contrast is needed. The controllable imaging system we have developed minimizes risks from constitutive reporter gene overexpression and facilitates tumor cell monitoring *in vitro* and *in vivo*.

## INTRODUCTION

Molecular imaging to visualize biological and pathological processes includes various modalities, such as optical imaging [1, 2], radionuclide imaging [3–5] and magnetic resonance imaging (MRI) [6–8]. Optical imaging modalities are limited to studying small animals or surface tissues [9–11]. Furthermore, while radionuclide imaging can be used in deep tissue, it achieves poor spatial and temporal resolution [12, 13]. On the other hand, MRI is a noninvasive technique that delivers superior spatial resolution and unlimited tissue penetration [6–8, 14, 15].

Early limitations to the sensitivity and specificity of molecular imaging with MRI [16] were overcome by the use of contrast agents, such as superparamagnetic iron oxide particles (SPIOs) with high relaxivity [17–21]. However, labeling cells with SPIOs to visualize cell proliferation and migration is not suitable for long-term cell tracking because the SPIOs become diluted as cells proliferate [16, 22, 23]. Additionally, particles released from apoptotic or lytic cells could be phagocytosed by macrophages in nearby tissues, leading to incorrect interpretations of MRI results [18, 24]. Furthermore, using SPIOs to label cells can affect cell differentiation [19–21]. A solution to these shortcomings is to genetically modify cells to express an MRI reporter gene.

MRI reporter genes allow studying dynamic cellular processes over an extended period of time because reporter genes can be integrated into the host genome [25]. This method is more stable than those that rely on particle retention and is less susceptible to signal loss through cell division [26, 27]. As iron is an essential element and can be metabolized in the body, iron metabolism genes have become targets of manipulation to provide MRI reporters, such as the transferrin receptor [28], magA [29, 30] and ferritin [31–34]. Among these candidates, ferritin is more attractive due to its efficient iron uptake and high transverse relaxation rate (R_2_). Native ferritin is a heteropolymer composed of 24 heavy and light subunits; of these, ferritin heavy chain (FTH1) exhibits more ferroxidase activity, promoting iron incorporation [16, 35–37]. As an MRI reporter, FTH1 alone or in combination with ferritin light chain has been used to monitor cancer cells [38–40], stem cells [22, 41, 42], fibroblasts [43] and dendritic cells [44].

Effective reporters should not be toxic to cells. Some studies on the safety of FTH1 as an MRI reporter have yielded concerning results. For example, the growth of HeLa [45] and nasopharyngeal carcinoma (NPC) [16] cells, was significantly reduced due to FTH1 overexpression. Safe and effective MRI reporter gene systems are in high demand for preclinical and clinical applications. Thus, we propose the use of FTH1 coupled with a Tet-On inducible system to achieve transgene activation with noninvasive, spatiotemporal control. There have been no previous reports on longitudinal monitoring of cancer cells *in vivo* using FTH1 in an inducible manner. This approach could minimize toxicity from constitutive reporter overexpression and be used to monitor cells via MRI only when needed. In this study, we combine the doxycycline (Dox)-sensitive Tet-On system and the reporter gene FTH1 to produce a Dox-triggered genetic reporter system, and we apply this system to longitudinally track implanted SK-N-SH cells via MRI. Our aim was to achieve a reporter gene capable of being switched “on” or “off” to image and monitor cell grafts *in vivo*.

## RESULTS

### Inducible expression of FTH1 in SK-N-SH-FTH1 cells

**Figure 1:**
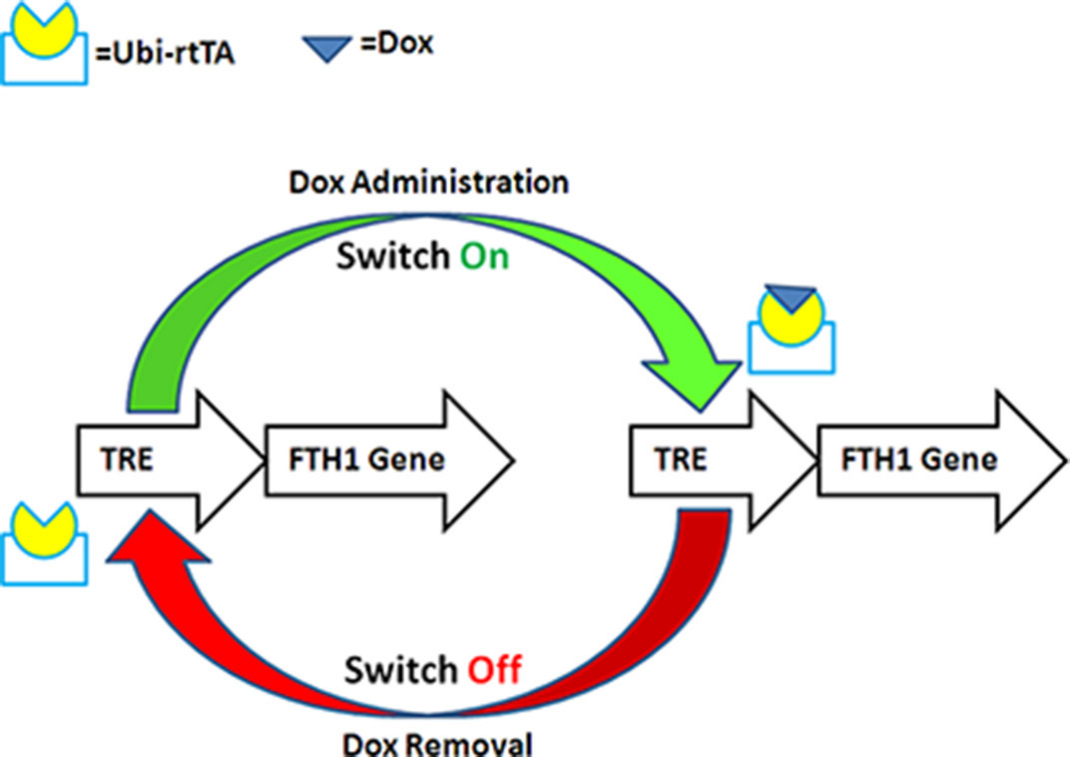
Schematic illustration of Dox-controlled FTH1 expression FTH1 expression was controlled by a tetracycline-responsive element (TRE). With Dox administration, the reverse tetracycline-controlled transactivator (rtTA), regulated by ubiquitin (Ubi), activated the TRE and switched “on” FTH1 expression. With Dox withdrawal, the TRE was not activated by rtTA, and FTH1 expression was switched “off.”

The reporter gene FTH1 was cloned into the pLenti-Tet-on-MCS-3Flag-Puro vector, and the resulting vector, pLV-Tet-FTH1, could simultaneously express Flag and FTH1 under the control of an inducible Dox-responsive promoter (Figure 1). SK-N-SH cells expressing FTH1 under the control of a Tet-On switch (SK-N-SH-FTH1) were successfully established by transfection with LV-Tet-FTH1 and clonal selection using puromycin.

**Figure 2:**
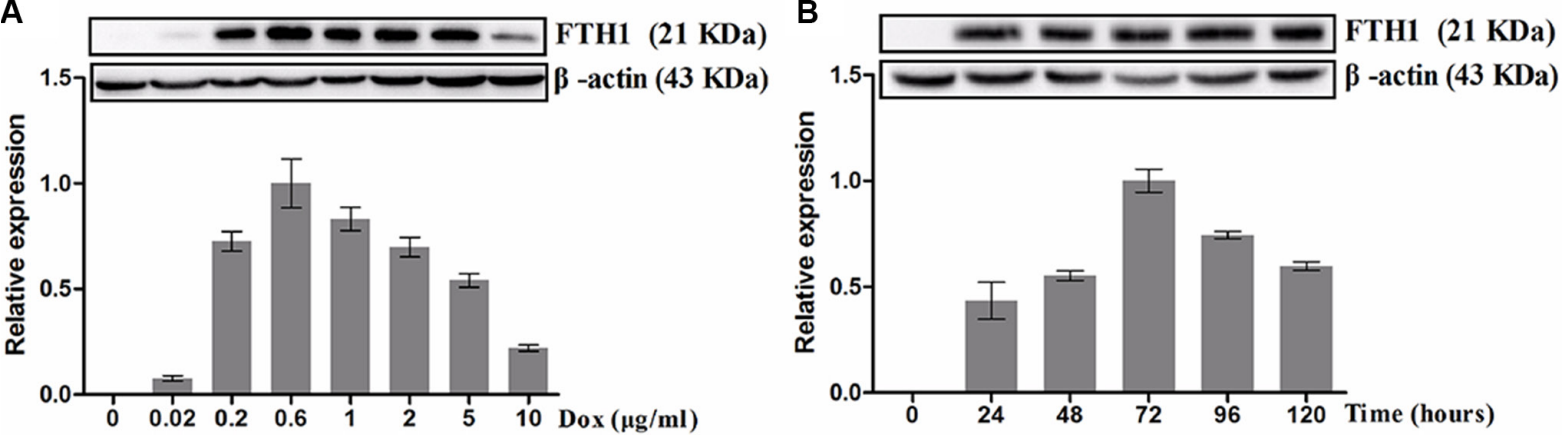
Western blot analysis of Dox-inducible FTH1 expression A. Dox dose-dependent FTH1 expression: SK-N-SH-FTH1 cells were cultured with different concentrations of Dox. With increasing Dox concentrations, FTH1 expression increased, peaking at 0.6 μg/ml Dox; however, FTH1 expression decreased with higher Dox concentrations. B. Time-dependent FTH1 expression: SK-N-SH-FTH1 cells were cultured for different durations in medium containing 0.6 μg/ml Dox. FTH1 expression reached its peak at 72 h and then decreased over time.

**Figure 3:**
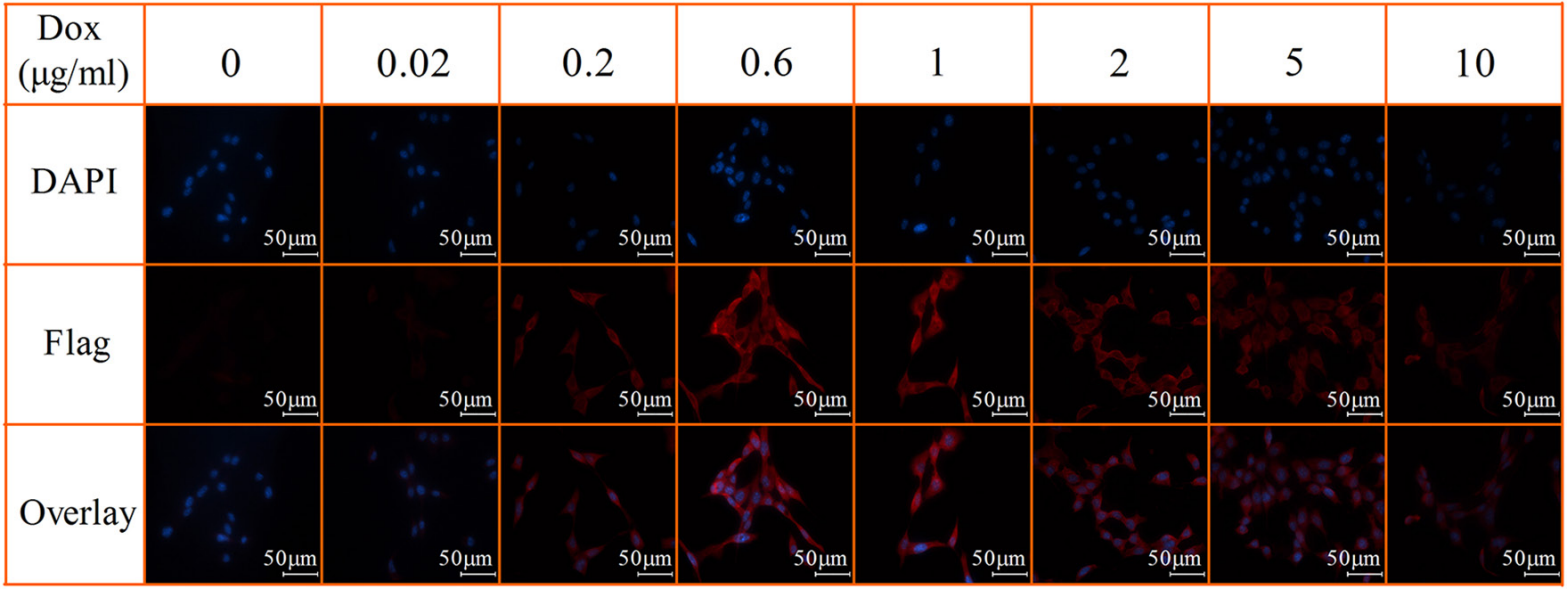
Immunofluorescent staining of Dox-inducible Flag expression SK-N-SH-FTH1 cells were cultured for 72 h in various concentrations of Dox. Immunostaining with a flag-specific antibody showed that flag expression (red fluorescence) was significantly stronger in SK-N-SH-FTH1 cells after being exposed to 0.6 μg/ml Dox for 72 h, which was consistent with the western blot results. Cell nuclei were stained with DAPI (blue). The scale bars all represent 50 μm.

To assess the dose-dependent expression of FTH1 *in vitro*, SK-N-SH-FTH1 cells were grown for 72 h in medium containing different concentrations of Dox. The western blot results showed that FTH1 expression increased gradually with increasing Dox concentrations, peaked at 0.6 μg/ml Dox, and then decreased with increasing Dox concentrations (Figure 2A). To assess the time-dependent expression of FTH1 *in vitro*, SK-N-SH-FTH1 cells were treated with Dox for various durations. The results demonstrated that FTH1 expression increased gradually over the course of 72 h, and then declined gradually (Figure 2B). Similar results were observed after immunofluorescence staining with a flag-specific antibody (Figure 3). The flag expression (red fluorescence) observed in SK-N-SH-FTH1 cells induced with 0.6 μg/ml Dox for 72 h was significantly stronger than that observed in other groups. Additionally, the expression of neither FTH1 nor Flag was detected in SK-N-SH-FTH1 cells without Dox induction. These results indicated that the optimal induction conditions (0.6 μg/ml Dox for 72 h) could be used for achieving maximal FTH1 expression in SK-N-SH-FTH1 cells.

### Cellular MRI contrast generated by the inducible expression of FTH1

**Figure 4:**
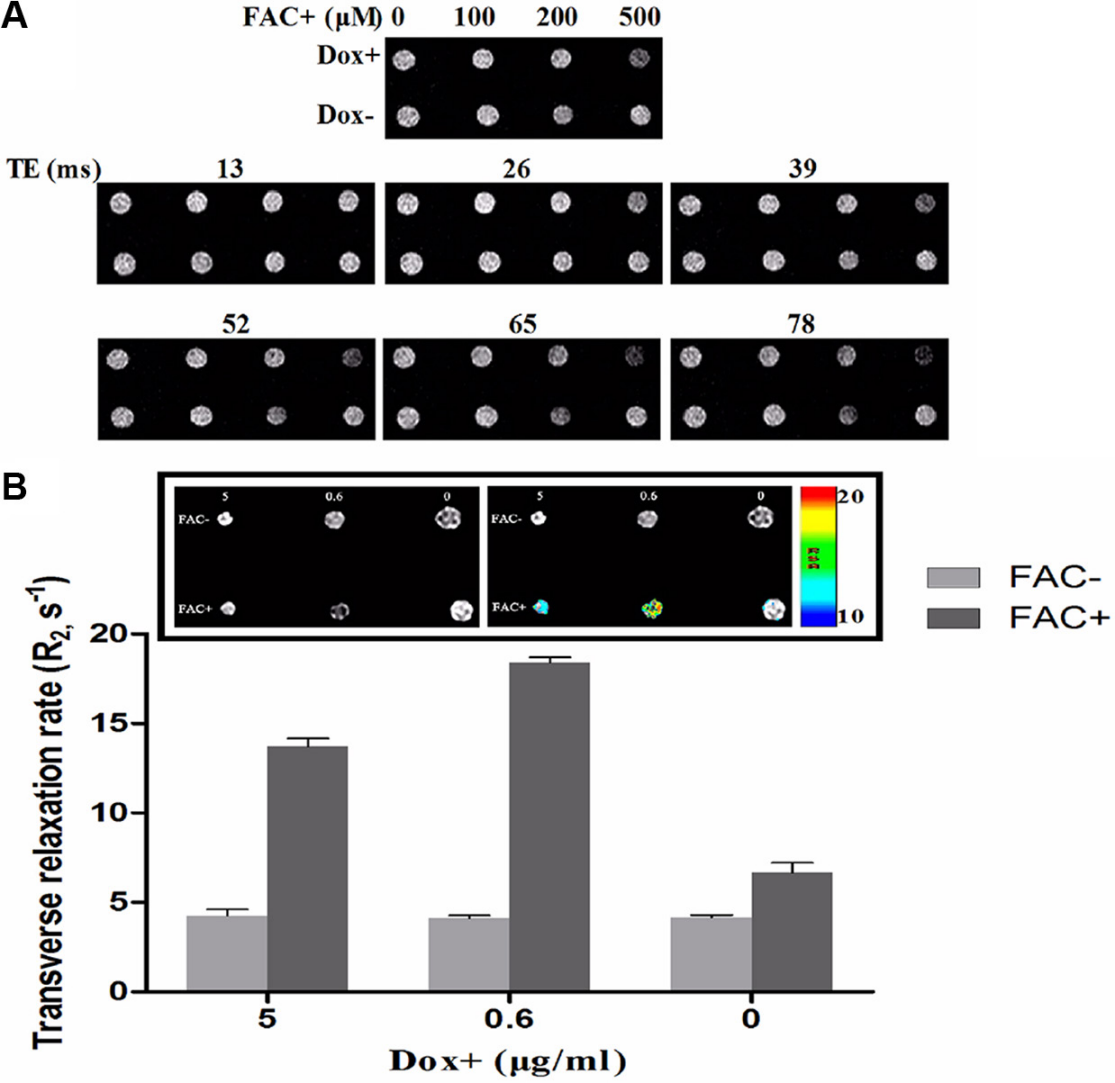
MRI contrast of cell pellets generated by inducible FTH1 expression (**A**) SK-N-SH-FTH1 cells were incubated in medium containing various concentrations of FAC with or without Dox. Without Dox (Dox-), T_2_-weighted MRI did not show significant signal changes. However, treatment with Dox (Dox+) resulted in decreasing signal intensity with increasing iron concentrations, and with 500 μM of FAC, the MRI signal was significantly decreased. (**B**) SK-N-SH-FTH1 cells were incubated with different concentrations of Dox in the presence or absence of 500 μM FAC. R_2_ values obtained from multi-echo measurements were higher in SK-N-SH-FTH1 cells than in the other cells after treatment with 0.6 μg/ml Dox and 500 μM FAC.

FTH1 expression may increase the ability of cells to internalize and store ferric ions, resulting in decreased MRI contrast. T_2_-weighted images and R_2_ values were obtained from SK-N-SH-FTH1 cells treated under different conditions. After treatment with the same Dox concentration (0.6 μg/ml) for 72 h, the MRI signal was dependent on the dose of ferric ammonium citrate (FAC). With 500 μM FAC, there was a notable hypointensity in the obtained T_2_-weighted images (Figure 4A). Further increases in the FAC concentration were not examined due to the potential risks of higher FAC concentrations. To determine whether different levels of FTH1 expression in SK-N-SH-FTH1 cells alter R_2_ values, the cell pellets were treated under six conditions. In the absence of FAC, the R_2_ values were very low regardless of the Dox treatment. However, in the presence of 500 μM FAC, the R_2_ values depended on Dox concentration. When the SK-N-SH-FTH1 cells were treated with 0.6 μg/ml Dox and 500 μM FAC, the R_2_ value was significantly higher than that of all other groups. When the SK-N-SH-FTH1 cells were treated with 5 μg/ml Dox and 500 μM FAC, the R_2_ value was higher than that in the corresponding control but lower than that resulting from 0.6 μg/ml Dox and 500 μM FAC. When the SK-N-SH-FTH1 cells were treated with 500 μM FAC in the absence of Dox, the R_2_ value was slightly higher than that in the corresponding control but lower than that in the groups treated with both Dox and FAC. These results indicated that iron availability and FTH1 expression promote the generation of R_2_ (Figure 4B).

### Intracellular iron accumulation following induced FTH1 expression

**Figure 5:**
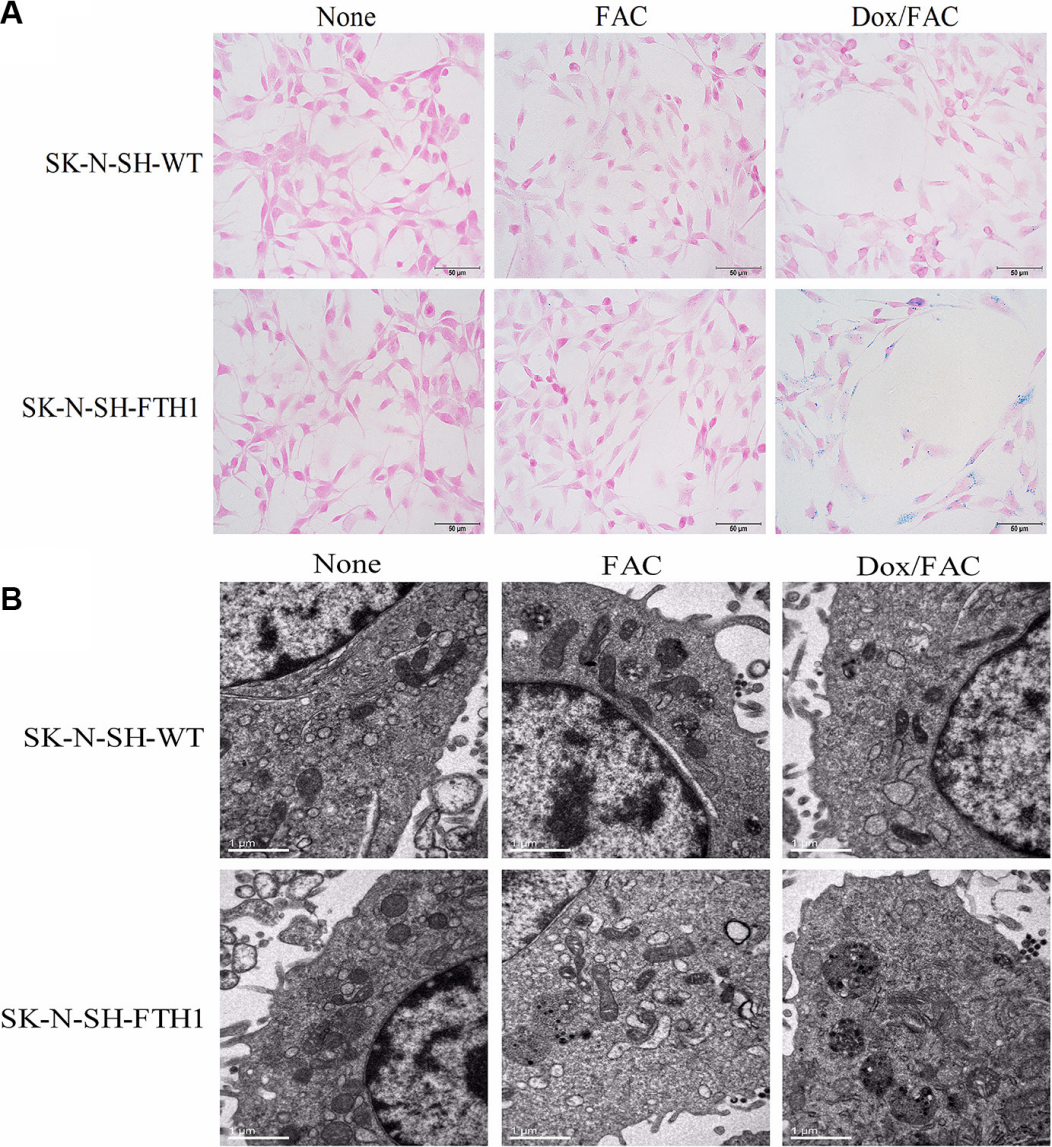
Intracellular iron accumulation in SK-N-SH-FTH1 and SK-N-SH-WT cells (**A**) Prussian blue staining revealed blue iron particles distributed throughout the cytoplasm of SK-N-SH-FTH1 cells but few particles dispersed within SK-N-SH-WT cells upon Dox induction and FAC supplementation (Dox/FAC, 0.6 μg/ml Dox and 500 μM FAC). Few blue particles were present within the SK-N-SH-WT and SK-N-SH-FTH1 cells treated only with FAC (FAC group). No blue particles were detected in SK-N-SH-FTH1 or SK-N-SH-WT cells in the absence of both Dox and FAC (None group). (**B**) The TEM results, which showed black iron particles accumulated in cytoplasmic vacuoles, were consistent with those of Prussian blue staining. The scale bars represent 50 μm (A) and 1 μm (B).

The intracellular iron accumulation resulting from FTH1 expression was assayed using Prussian blue staining and transmission electron microscopy (TEM). Prussian blue staining revealed large, dense, blue particles of accumulated iron distributed throughout the cytoplasm of SK-N-SH-FTH1 cells but only a small amount dispersed in SK-N-SH-WT cells cultured in the same Dox- and FAC-supplemented medium (Dox/FAC group, 0.6 μg/ml Dox and 500 μM FAC). Few blue particles were present within the SK-N-SH-WT and SK-N-SH-FTH1 cells treated only with FAC (FAC group). No blue particles were detected in SK-N-SH-FTH1 or SK-N-SH-WT cells in the absence of Dox and FAC (None group) (Figure 5A). The TEM results were consistent with those of Prussian blue staining; the iron appeared as dense black particles accumulating in cytoplasmic vacuoles (Figure 5B).

### Effects of FTH1 expression on SK-N-SH cell proliferation

**Figure 6:**
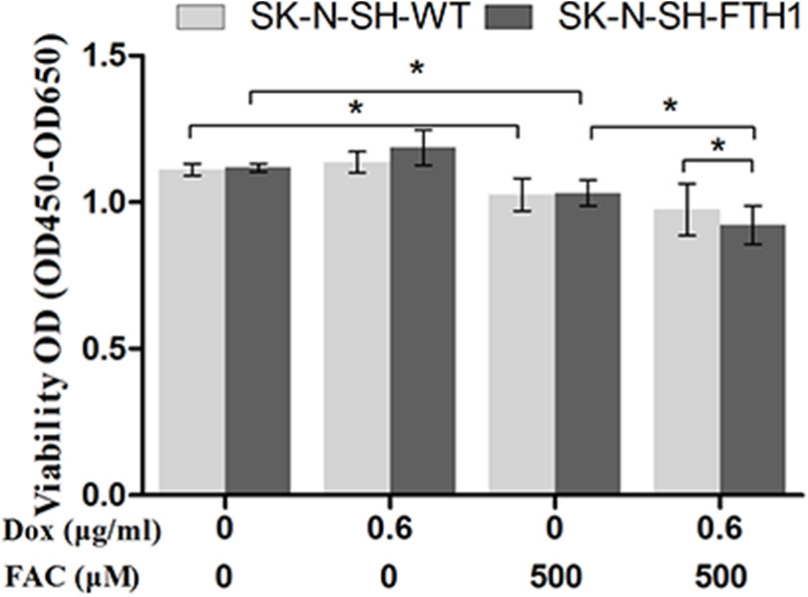
Cell proliferation analysis To evaluate the impact of FTH1 expression and/or iron accumulation on cells, the proliferation of SK-N-SH-FTH1 and SK-N-SH-WT cells incubated in a 96-well plate was examined using a CCK-8 assay. In the absence of FAC, FTH1 overexpression did not interfere with SK-N-SH cell proliferation *in vitro*. However, a relatively high dose (500 μM) of FAC inhibited the growth of SK-N-SH-WT and SK-N-SH- FTH1 cells. Interestingly, the cells expressing FTH1 were more sensitive to FAC.

To assess whether FTH1 transgene expression or iron accumulation inhibited SK-N-SH cell proliferation, a Cell Counting Kit 8 (CCK-8) assay was used to assess cell proliferation over a 72-h period (Figure 6). Without FAC supplementation, no significant differences were found in cell proliferation between SK-N-SH-WT and SK-N-SH-FTH1 cells, regardless of Dox supplementation. However, a relatively high dose (500 μM) of FAC inhibited the growth of both SK-N-SH-WT and SK-N-SH-FTH1 cells, regardless of treatment with 0.6 μg/ml Dox. Interestingly, in the presence of 500 μM FAC, SK-N-SH-FTH1 cells expressing FTH1 were more affected than both cells not expressing FTH1 and SK-N-SH-WT cells (*P* < 0.05). These results suggested that while the cells expressing FTH1 were more sensitive to FAC, the high iron concentration exerted side effects on both SK-N-SH-FTH1 and SK-N-SH-WT cells, regardless of FTH1 expression.

### *In vivo* MRI of cell grafts with inducible FTH1 expression

**Figure 7:**
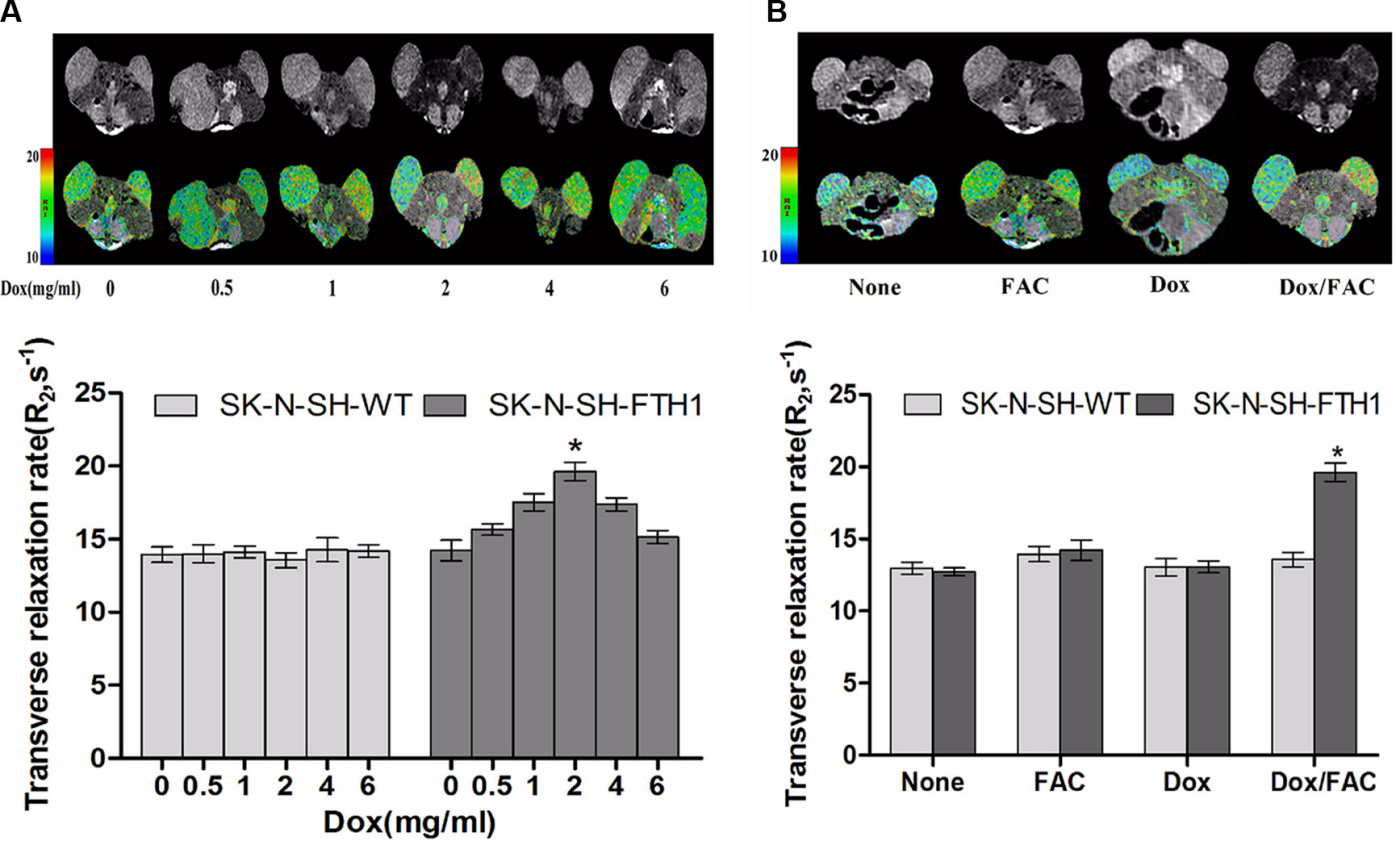
MRI validation of dose-dependent FTH1 expression in mouse tumors from subcutaneously inoculated cell grafts (**A**) SK-N-SH cells were inoculated into the hind limbs of nude mice (SK-N-SH-FTH1 cells in the left hind limb, corresponding to the right side of the images; SK-N-SH-WT cells in the right hind limb, corresponding to the left side of the images). MRI scans showed that compared with the SK-N-SH-WT grafts, the negative contrast of the SK-N-SH-FTH1 grafts increased gradually with increasing concentrations of Dox, a marked hypointensity was observed with 2 mg/ml Dox, and contrast decreased with higher Dox concentrations. There were no differences between the two types of cell grafts when the Dox concentration was 6 μg/ml. The R_2_ map and MRI results were similar. (**B**) The R_2_ values of SK-N-SH-FTH1 and SK-N-SH-WT cell grafts treated only with 5 mg/ml FAC (FAC group) for 5 days were slightly higher than those of the negative controls (None group), but far lower than that of the SK-N-SH-FTH1 cell grafts treated with 2 mg/ml Dox and 5 mg/ml FAC (Dox/FAC group). There were no significant differences between the SK-N-SH-FTH1 and SK-N-SH-WT cell grafts treated only with 2 mg/ml Dox (Dox group) for 5 days.

We conducted two sets of experiments to assess changes in MRI signal intensity with induced FTH1 expression *in vivo*. Dose-dependent FTH1 expression was determined in the first study. The MRI scans showed that compared with the SK-N-SH-WT grafts, the negative contrast in the SK-N-SH-FTH1 grafts was increased slightly at a Dox concentration of 1 mg/ml and was more intense at 2 mg/ml, but the contrast decreased with higher Dox concentrations. Accordingly, the R_2_ maps demonstrated increased R_2_ values in SK-N-SH-FTH1 grafts treated with 2 mg/ml Dox, but these values decreased with increasing Dox concentrations. In addition, there were no differences between the two types of cell grafts when the concentration of Dox was 6 μg/ml (Figure 7A). Furthermore, the R_2_ values of the SK-N-SH-FTH1 and SK-N-SH-WT grafts treated for 5 days only with 5 mg/ml FAC (FAC group) were slightly higher than those of the negative control grafts (None group) but far less than that of SK-N-SH-FTH1 grafts in the Dox/FAC group. We also found that there were no significant differences in R_2_ values between the two cell grafts treated only with 2 mg/ml Dox (Dox group) for 5 days (Figure 7B).

**Figure 8:**
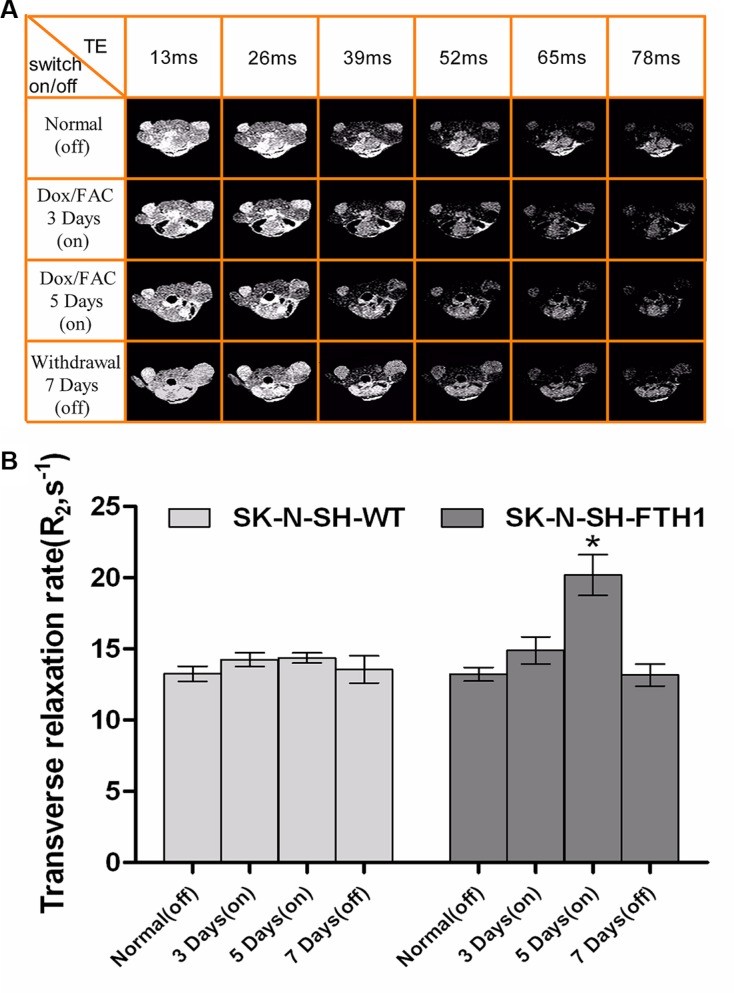
*In vivo* MRI results of a cell graft with (“on”) and without (“off”) induced FTH1 expression (**A**) A multi-echo MRI sequence of a single mouse that completed the longitudinal series of MRI scans, showing remarkable contrast between the SK-N-SH-FTH1 (left hind limb, corresponding to the right side of the images) and SK-N-SH-WT (right hind limb, corresponding to the left side of the images) cell grafts in the tumor-bearing nude mice when FTH1 was induced (“on”) by 2 mg/ml Dox and 5 mg/ml FAC for 5 days while no contrast was observed when FTH1 was not induced (“off”). When Dox was withdrawn for 7 days, the signal intensity was similar for both cell graft types. (**B**) The R_2_ values of SK-N-SH-FTH1 cell grafts treated with 2 mg/ml Dox and 5 mg/ml FAC for 5 days (5 days “on”) was higher than those found for other conditions (“off”, 3 days “on” and 7 days “off”). For SK-N-SH-WT cell grafts, there were no differences in the R_2_ values among the four conditions (normal “off”, 3 days “on” 5 days “on”, and 7 days “off”).

The second study showed that the MRI contrast of the SK-N-SH-FTH1 cell grafts could be generated or extinguished by switching the FTH1 expression “on” or “off” by controlling the administration of Dox. No differences in the MRI signal were observed between the SK-N-SH-FTH1- and the SK-N-SH-WT-derived tumors when Dox was not administered for 14 days after transplantation, i.e., after the reporter was switched “off.” Subsequently, FTH1 expression could be induced *in vivo*, i.e., switched “on,” by giving the mice 2 mg/ml Dox and 5 mg/ml FAC in their drinking water. On the 5th day, the MRI scans revealed significantly decreased MRI signals in the SK-N-SH-FTH1-derived tumors compared with the SK-N-SH-WT-derived tumors. After 7 days of withdrawal from Dox/FAC, i.e., when the reporter was switched “off,” signals from the region of the SK-N-SH-FTH1 grafts returned to baseline levels, similar to those of the SK-N-SH-WT grafts (Figure 8A). The R_2_ measurements were similar to those of the MRI signals. In the SK-N-SH-FTH1 cell grafts, the R_2_ values were significantly higher after 5 days of Dox/FAC treatment (5 days of being switched “on”) than after the other treatments (i.e., being switched “off,” 3 days of being switched “on,” and 7 days of being switched “off”). In the SK-N-SH-WT cell grafts, no differences in R_2_ values were observed regardless of the reporter being switched “on” or “off” (Figure 8B).

### Histological validation of FTH1 expression

**Figure 9:**
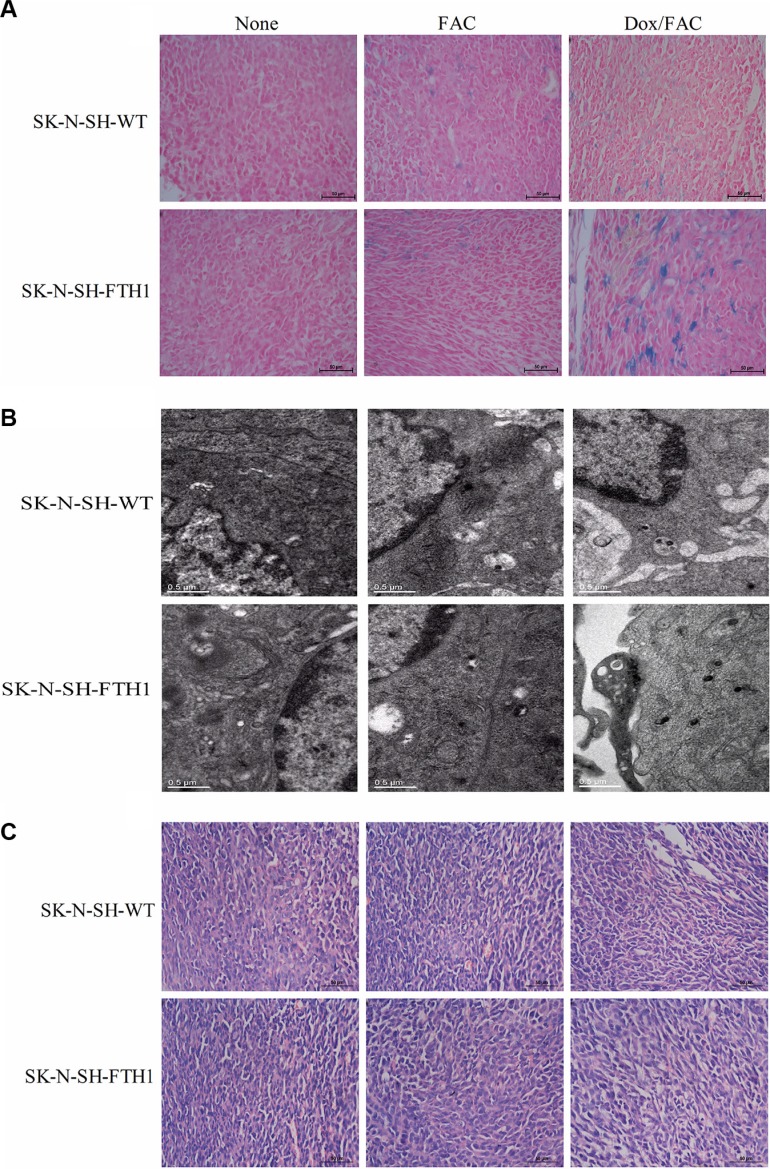
*Ex vivo* histological validation of FTH1 expression induced in subcutaneous SK-N-SH-WT and SK-N-SH-FTH1 tumors (**A**) Prussian blue staining showed numerous blue-positive cells in SK-N-SH-FTH1-derived tumors rather than in SK-N-SH-WT-derived tumors after Dox/FAC (2 mg/ml and 5 mg/ml) treatment. Very few blue-positive cells were observed in both tumor types when treated with FAC only. No iron accumulation occurred in either tumor type without FAC or Dox administration. (**B**) The TEM results, which showed iron present in the cytoplasm as dense black particles, were similar to those of Prussian blue staining. (**C**) No pathological changes were observed by H&E staining under FTH1 overexpression and/or iron supplementation. Scale bars: 50 μm (A), 0.5 μm (B), and 50 μm (C).

The *ex vivo* Prussian blue staining revealed more positively stained particles in the SK-N-SH-FTH1-derived tumors than in the SK-N-SH-WT-derived tumors after the administration of Dox/FAC (2 mg/ml and 5 mg/ml, respectively). Only small numbers of positive particles were detected in both SK-N-SH-FTH1- and SK-N-SH-WT-derived tumors after only FAC was administered. No iron accumulation was observed in either tumor type when neither FAC nor Dox were administered. In addition, the iron particles were not uniformly distributed in all tumors (Figure 9A). The TEM results, which showed iron present in the cytoplasm as dense black particles, were similar to those of Prussian blue staining (Figure 9B). The hematoxylin and eosin (H&E)-stained histological sections showed that the tumors were highly vascularized, and no visible pathological differences were associated with FTH1 expression and/or iron supplementation (Figure 9C).

## DISCUSSION

In this study, we successfully applied the Tet-On inducible FTH1 reporter system for the longitudinal, *in vivo* monitoring of implanted cell grafts. With this innovative reporter gene imaging system, we could not only track cancer cells via MRI as needed but also minimize the potential adverse impacts of continuous FTH1 overexpression and iron accumulation on cell growth. This noninvasive, reproducible and controllable imaging tool could also be used with other cell lines, thereby furthering cellular therapy strategies.

The application of FTH1 as a genetic reporter poses the risks of long-term gene overexpression and cellular iron accumulation. To date, consensus is lacking regarding the effect of FTH1 overexpression on cells. While some reports showed that iron-independent FTH1 overexpression did not alter the growth rate of various types of cells [8, 18, 38, 46, 47], others showed evidence of deleterious side effects [16, 45]. In addition, the study by Feng *et al.* [16] showed that moderate FTH1 expression did not reduce NPC cell proliferation regardless of iron administration. However, maximal FTH1 expression decreased the cell growth rate in the absence of iron supplementation. These results suggested that the effects of FTH1 overexpression depend on cell type and the FTH1 levels, as well as on the presence or absence of iron. Here, we showed that FTH1 expression in SK-N-SH cells did not inhibit cell proliferation in the absence of supplemental iron, while the opposite effect was seen with supplemental iron. With 500 μM FAC, the growth rate of SK-N-SH-FTH1 cells expressing FTH1 was more inhibited than that of SK-N-SH-WT cells (*P* < 0.05). This finding indicated that FTH1 expression leads to iron accumulation and then increases the levels of reactive oxygen species, which could induce cell injury or apoptosis [48]. Therefore, while the search for an ideal reporting system continues, such an inducible genetic reporter as “Tet-FTH1” provides a unique tool for the long-term longitudinal tracking of cell grafts.

In this study, we successfully demonstrated the switch “on” or “off” function of a genetic reporter for tracking cancer cells using MRI. When the cell grafts needed to be assessed, the Tet-On switch was turned on by the administration of Dox. Then, the reporter gene FTH1 located downstream of Tet-On was expressed, and iron accumulated inside the cells, generating MRI contrast. In contrast, when the implanted cells did not need to be assessed, the Tet-On switch was turned off by withdrawing Dox, thereby silencing the reporter FTH1 gene. By using the Tet-On switch, unnecessary FTH1 expression and intracellular iron accumulation were avoided. In consideration of animal welfare, due to the rapid development of the SK-N-SH-FTH1 cell-derived tumors in the late stage, we performed only four MRI scan series with “on” and “off” FTH1 expression as a proof of concept. When the MRI scans revealed significantly decreased signals in the SK-N-SH-FTH1-derived tumors compared with the SK-N-SH-WT-derived tumors on the 5th day of induced FTH1 expression, the Tet-On switch was turned off. Thus, 5 days is not necessarily the optimal duration of *in vivo* Dox induction for FTH1 expression in cancer cells. On the other hand, although the MRI contrast recovered after 7 days of Dox/FAC withdrawal, 7 days is not necessarily the shortest time for MRI signal recovery. The future use of less tumorigenic, lineage-specific cell types, such as NPC or stem cells, will allow for long-term longitudinal examination of cell grafts with more iterations of induced or inhibited FTH1 expression followed by MRI. Non-tumorigenic cell types will also enable the investigation of FTH1 sensitivity and detection thresholds, which were not addressed in the current study.

Our results showed that Dox-induced FTH1 expression is dose- and time-dependent. We detected no FTH1 expression in SK-N-SH-FTH1 cells in the absence of Dox, suggesting negligible background expression for the Tet-On system and controllable transgene expression. Furthermore, FTH1 expression increased with increasing Dox concentrations and peaked *in vitro* at 0.6 μg/ml Dox. This differs from the results of our previous study, in which FTH1 expression in C3H10T1/2 cells reached a peak at a 0.2 μg/ml Dox [8]. This difference could be due to the varying susceptibility of different cell lines to Dox. In addition, here we observed variations in the FTH1 expression pattern, iron content, and contrast signals of SK-N-SH-FTH1 cells and SK-N-SH-FTH1-derived tumors. According to our cell pellet phantom study, induced FTH1 expression in SK-N-SH-FTH1 cells decreased the MRI signal upon culture with 0.6 μg/ml Dox for 72 h while SK-N-SH-FTH1-derived tumors exhibited only slightly decreased signals, even after 5 days of treatment with 2 mg/ml Dox. We speculate that this discrepancy might result from a failure of *in vitro* cultures to accurately reflect the complexity of cancer cell biology *in vivo*. In addition, the presence of Dox or FAC in cell culture medium could directly affect each cell. On the other hand, the responses of individual experimental animals cannot be as accurately controlled *in vivo*. In our study, Dox and FAC slowly and weakly affected SK-N-SH-FTH1-derived tumors, possibly due to the first-pass effect and time-consuming blood circulation. In addition, as with other tumor types [9], the blood supply may not have been evenly distributed in these tumors, yielding a variable distribution of Dox and FAC. Furthermore, variations in growth rate and differentiation stage could also elicit variable responses. As a result, heterogeneous relaxation rates in tumors may have resulted from variable degrees of induced FTH1 expression and iron accumulation.

While T_2_*-weighted imaging is more sensitive to the effects of iron, T_2_-weighted imaging presumably provides a more accurate representation of the spatial distribution of cells [30]. In this study, we applied T_2_-weighted imaging rather than T_2_*-weighted imaging to visualize SK-N-SH-FTH1 cells and SK-N-SH-FTH1-derived tumors using a 3.0-T MRI system. As expected, MRI contrast was effectively detected due to inducible FTH1 expression with iron supplementation under optimal conditions in both the SK-N-SH-FTH1 cells and the grafted tumors. However, in this study, FTH1 expression without iron supplementation did not affect the MRI signals. This result was inconsistent with other studies reporting iron redistribution and altered MRI contrast upon ferritin expression, even without changes in total iron content [22, 23, 38]. Without exception, these studies employed MRI systems with field strengths of 7 T or higher, which are much more magnetically sensitive than our 3-T MRI system. FTH1 overexpression enhances MRI contrast by two mechanisms: 1) extracellular iron uptake and 2) changes in the relaxation rate of intracellular iron redistributed in ferritin pools. A 3.0-T MRI system might lack the sensitivity to detect changes in endogenous iron resulting from FTH1 expression. With this system, administration of exogenous iron was required to ensure the generation of MRI contrast. As the transverse relaxivity rate increases in parallel with the magnetic field strength [38], we postulated that performing MRI with higher magnetic fields could more effectively visualize FTH1-tagged cells or grafts by providing improved imaging quality and resolution, even without exogenous iron administration. This strategy could extend the applicability of the Tet-On inducible reporter gene system and accelerate its bench-to-bedside translation.

In addition to requiring supplementation with exogenous iron, our study suffered from other limitations. For example, very few animals were used for *in vivo* experiments and our R_2_ evolution analysis of the switch system to determine optimal “on” and “off” points could be improved by including more time points. Nonetheless, we have verified the feasibility of using FTH1 as a reporter under the regulation of a Tet-On system for mapping gene expression *in vivo* via MRI. Our controllable imaging system may facilitate tumor cell monitoring and allow tracking of other cell types *in vitro* and *in vivo*. In addition, combining the Tet-On inducible imaging system with specific cell promoters, such as tumor or neural cell-specific promoters, could efficiently reduce the side effects of reporter gene overexpression and accurately identify and monitor specific cell types.

## MATERIALS AND METHODS

### Inducible ferritin expression vector design

Human FTH1 (accession number BC000857) cDNA was generated via polymerase chain reaction (PCR) amplification using the following primers: forward, AACCGTCAGATCGCACCGGTGCCACCATGACGAC CGCGTCCACCTC; and reverse, TCCTTGTAGTCC ATGAATTCGCTTTCATTATCACTGTCTC. Then, this cDNA was subcloned into the multiple cloning site of a tetracycline-inducible lentiviral vector, pLenti-Tet-MCS-3Flag-Puro (GeneChem Co., Ltd., Shanghai, China), using *AgeI* and *EcoRI*, which generated the pLenti-Tet-FTH1-3Flag-Puro plasmid vector (pLV-Tet-FTH1). A replication-defective lentivirus expressing Tet-FTH1 (LV-Tet-FTH1) was produced by cotransfecting the plasmids pLV-Tet-FTH1, pHelper 1.0 (composed of structural genes for virion assembly; GeneChem Co., Ltd., Shanghai, China) and pHelper 2.0 (GeneChem Co., Ltd., Shanghai, China) into 293T packaging cells (Invitrogen, Carlsbad, CA, USA). Culture medium was collected at 48 h post-transfection. The resulting lentivirus LV-Tet-FTH1 constitutively coexpressed FTH1 and Flag via the Tet-On inducible promoter system; samples were distributed into multiple tubes and stored at −80°C for further experiments.

### Cell maintenance

Wild-type human neuroblastoma cells (SK-N-SH-WT; obtained from Chongqing Pediatric Medical Research Institute, Chongqing, China) were seeded and cultured in complete medium consisting of Dulbecco's modified Eagle's medium (Beyotime, Nanjing, Jiangsu, China) with 10% FBS (Beyotime, Nanjing, Jiangsu, China) and 0.5% penicillin/streptomycin in a humidified atmosphere containing 5% CO_2_ at 37°C. Upon reaching 90% confluency, the cells were treated with 0.25% trypsin- ethylenediaminetetraacetic acid (EDTA; Beyotime, Nanjing, Jiangsu, China), dissociated into single-cell suspensions, and subcultured at a split ratio of 1:3 to 1:5.

### Establishment of clonal transgenic SK-N-SH cells

To generate transgenic SK-N-SH cells expressing Flag-tagged FTH1 protein under the control of a Tet-On switch, 30–40% confluent SK-N-SH-WT cells were transfected with the LV-Tet-FTH1 lentivirus in the presence of 10 μg/ml polybrene (Beyotime, Nanjing, Jiangsu, China). At 72 h post-transfection, 8 μg/ml puromycin (Beyotime, Nanjing, Jiangsu, China) was used to select a clonal cell line (SK-N-SH-FTH1) for use in subsequent experiments.

### Western blot analysis

To assess the dose-dependent expression of FTH1, SK-N-SH-FTH1 cells were grown for 72 h in medium containing various concentrations (0, 0.02, 0.2, 0.6, 1, 2, 5 and 10 μg/ml) of Dox (Santa Cruz, Dallas, TX, USA), which is a tetracycline derivative. To examine the time-dependent expression of FTH1, SK-N-SH-FTH1 cells were cultured for different durations in the presence of an optimal Dox concentration. After treatment, the cells were collected and prepared for western blot analysis according to previously reported protocols [8]. In brief, the cells were lysed in lysis buffer (Sigma-Aldrich, St. Louis, MO, USA) containing protease inhibitors, phosphatase inhibitors and 100 mM phenylmethylsulfonyl fluoride. The total protein concentration in the samples was determined using the bicinchoninic acid (BCA; Sigma-Aldrich, St. Louis, MO, USA) method. Then, 30 μg of protein from each sample was loaded onto 12% sodium dodecyl sulfate-polyacrylamide gels and transferred onto polyvinylidene difluoride membranes (Millipore, Billerica, Madrid, Spain). To detect human FTH1, we blocked the membranes for 1 h with 5% bovine serum albumin (BSA; Beyotime, Nanjing, Jiangsu, China). Then, the membranes were incubated overnight at 4°C with primary antibodies specifically recognizing FTH1 (rabbit anti-FTH1, 1:1,000; Abcam, Cambridge, MA, UK) or β-actin (mouse anti-β-actin; Nanjing Zoonbino Biotechnology Co., Ltd., Nanjing, Jiangsu, China). The membranes were subsequently washed with Tris-buffered saline containing Tween-20 (TBST) and incubated for 2 h with secondary antibodies (anti-rabbit 1:5,000, Abgent, San Diego, CA, USA; anti-mouse 1:1000, GenScript, Nanjing, Jiangsu, China). The resulting protein bands were viewed using an enhanced chemiluminescence kit (Sigma-Aldrich, St. Louis, MO, USA). The relative expression of FTH1 in the SK-N-SH-FTH1 cells was normalized to human β-actin expression and analyzed semi-quantitatively from band intensity.

### Immunofluorescence staining

To indirectly determine the expression levels of FTH1 by immunostaining, Flag-expressing SK-N-SH-FTH1 cells were treated under the same conditions as those of the western blot analysis. After treatment, the cells were rinsed with phosphate-buffered saline (PBS), fixed with 4% paraformaldehyde, and thoroughly washed. The samples were permeabilized with 1% Triton X-100 in PBS, blocked with 5% BSA at 37°C for 30 min, and incubated overnight at 4°C with a primary antibody against Flag (mouse anti-Flag 1:200; Abgent, San Diego, CA, USA). The cells were then washed with PBS and incubated in the dark at room temperature with the secondary antibody (Cy3-conjugated anti-mouse, 1:1,000; Beyotime, Nanjing, Jiangsu, China). Subsequently, 4, 6-diamidino-2-phenylindole (DAPI; Beyotime, Nanjing, Jiangsu, China) was used to counterstain cell nuclei. After mounting coverslips, the samples were imaged using a fluorescence microscope (Nikon, Tokyo, Japan).

### MRI of cell pellets

To determine the suitable concentration of iron supplementation for producing cellular MRI contrast in the presence of FTH1 expression, SK-N-SH-FTH1 cells were cultured for 72 h in medium with various concentrations (0, 100, 200 and 500 μM) of FAC (Santa Cruz, Dallas, TX, USA) with or without Dox induction. Then, to explore the relationship between the FTH1 expression level and the R_2_ value, SK-N-SH-FTH1 cells were treated for 72 h with different concentrations (0, 0.6, and 5 μg/ml) of Dox in the presence or absence of 500 μM FAC. All treated cells were thoroughly washed with PBS to remove free FAC; then, the cells were trypsinized with EDTA, resuspended with PBS, and transferred into 600-μl Eppendorf tubes to prepare cell phantoms for *in vitro* MRI. The cells settled to the bottom of the tubes and formed loose pellets. A 3.0-T MRI scanner (Phillips, Eindhoven, Netherlands) with a knee coil was employed to image the cell phantoms. A multi-echo sequence was performed with the following parameters: repetition time (TR) = 2000 ms; echo time (TE) = 13–78 ms; step size = 13 ms (six-point T_2_ mapping), field of view (FOV) = 180 × 180 mm, image matrix = 512 × 512, and slice thickness = 1 mm. Reconstruction software installed on the post-processing work station was used to obtain the R_2_ color maps, from which the R_2_ values were measured.

### Prussian blue staining

Prussian blue staining was performed to assess intracellular iron accumulation and distribution. SK-N-SH-WT or SK-N-SH-FTH1 cells grown on coverslips were treated for 72 h under the following conditions: no Dox/no FAC (None group); no Dox/500 μM FAC (FAC group); or 0.6 μg/ml Dox/500 μM FAC (Dox/FAC group). After treatment, these cells were prepared for Prussian blue staining according to previously published protocols [8],

### TEM

SK-N-SH-WT and SK-N-SH-FTH1 cells were treated under the same conditions used for Prussian blue staining. After treatment, the cells were prepared for TEM according to previously published protocols [8]. An H-7500 transmission electron microscope (Hitachi, Tokyo, Japan) was used to visualize the intracellular iron accumulation.

### Assessment of cell growth

SK-N-SH cell growth was assessed using a CCK-8 assay (Beyotime, Nanjing, Jiangsu, China). SK-N-SH-WT and SK-N-SH-FTH1 cells were incubated in a 96-well plate and treated for 72 h with 0.6 μg/ml Dox, 500 μM FAC, or both. Relative changes compared with the untreated group were calculated for both cell lines.

### Xenograft tumor model

The animals used in this study were purchased from the Medical Laboratory Animal Department of Chongqing Medical University, and they were maintained in the experimental animal center of our institute. All animal experiments were performed strictly according to the policies approved by the Animal Experimental Ethics Committee of Chongqing Medical University. For inoculation, cells (6 × 10^6^ cells per limb) suspended in 100 μl of medium were subcutaneously injected into the hind limbs of 6-week-old male nude mice weighing 20–25 g. SK-N-SH-FTH1 cells were injected into the left hind limb, while SK-N-SH-WT cells were injected into the right hind limb. The xenograft tumors were allowed to grow for 2 weeks before the experiments began. The tumor-bearing mice were housed 1 per cage under a 12-hour light-dark cycle and controlled temperature (22°C). Over 2 weeks of observation, the drinking water intake of one animal was determined to be 5–8 ml per day. As such, when drug administration was needed, each mouse was given 5 ml (minimal quantity) of water per day to ensure total intake of the drug-supplemented water.

### *In vivo* MRI of cell grafts in mice

Two sets of experiments were carried out in this part of the study. The first experiment was conducted to determine the optimal concentration of Dox for inducing significant MRI contrast. Mice were randomly assigned to six experimental groups; three mice were included in each group. The mice were given water supplemented with various concentrations of Dox (0, 0.5, 1, 2, 4 or 6 mg/ml) and 5 mg/ml of FAC for 5 days. MRI scans were subsequently performed to determine the optimal Dox concentration. The untreated mice and the mice treated with only the optimal concentration of Dox or 5 mg/ml of FAC were also scanned as controls.

The second experiment was performed to determine whether MRI contrast could be switched “on” (with Dox) or “off” (without Dox) in the presence of iron supplementation while longitudinally monitoring grafted cells *in vivo*. Mice were fed a normal diet for 14 days (“off” status), followed by an MRI. Then, the mice were given water containing 2 mg/ml Dox and 5 mg/ml FAC (“on” status) and were scanned after 3 and 5 days. Finally, Dox and FAC were withdrawn for 7 days (“off” status), and the mice were scanned again to determine whether the MRI contrast had returned to the baseline.

For *in vivo* imaging, the animals were anesthetized by an intraperitoneal injection of pentobarbital sodium (30 mg/kg). A 3.0-T MRI system (Phillips, Eindhoven, Netherlands) with a knee coil was applied. Warm cotton was used to keep the animals warm during the MRI scan. Six-point T_2_ mapping was obtained by using a multi-echo spin-echo T_2_-weighted sequence. The scanning parameters were as follows: TR, 2000 ms; echo settings, 13 and 78 ms with a step size of 13 ms; matrix, 380 × 311; FOV, 160 × 160 mm; and horizontal slices, 1.2 mm thick. R_2_ values were measured from R_2_ color maps.

### Histological analysis

After the MRI studies, animals from the three groups (None: no Dox or FAC; FAC: no Dox and 5 mg/ml FAC; Dox/FAC: 2 mg/ml Dox and 5 mg/ml FAC) were sacrificed, and their tumors were harvested and fixed with ice-cold, 4% paraformaldehyde. Each tumor was embedded in paraffin and sectioned. Then, the 4-μm-thick paraffin sections were prepared for H&E staining to observe pathological changes. Prussian blue staining and TEM were also performed to visualize iron accumulation in the tumor tissues.

### Statistical analysis

All data are expressed as the mean ± standard deviation. Statistical Package for the Social Sciences version 13.0 (SPSS Inc., Chicago, IL, USA) was used for statistical analyses. One-way analysis of variance and the least significant difference method were used to examine differences among the groups. *P* values less than 0.05 were considered statistically significant.

## Abbreviations

MRI: magnetic resonance imaging
SPIOs: superparamagnetic iron oxide nanoparticles
R2: transverse relaxation rate
FTH1: ferritin heavy chain
NPC: nasopharyngeal carcinoma
Dox: doxycycline
Tet: tetracycline
PCR: polymerase chain reaction
LV-Tet-FTH1: lentivirus expressing Tet-FTH1
pLV-Tet-FTH1: pLenti-Tet-on-FTH1-3Flag-Puro
FBS: fetal bovine serum
PBS: phosphate-buffered saline
BCA: bicinchoninic acid
BSA: bovine serum albumin
TBST: Tris-buffered saline containing Tween-20
DAPI: 46-diamidino-2-phenylindole
FAC: ferric ammonium citrate
TR: repetition time
TE: echo time
FOV: field of view
CCK-8: Cell Counting Kit 8.
